# Declines in Activities in Daily Living of Older Adults with Sarcopenia Were Associated with Gait Speed

**DOI:** 10.3390/medicina62020263

**Published:** 2026-01-26

**Authors:** Ryo Sato, Yohei Sawaya, Tamaki Hirose, Takahiro Shiba, Lu Yin, Shuntaro Tsuji, Masahiro Ishizaka, Tomohiko Urano

**Affiliations:** 1 Integrated Facility for Medical and Long-Term Care, Care Facility for the Elderly “Maronie-en”, 533-4 Iguchi, Nasushiobara 329-2763, Japan; satoryo1220@gmail.com (R.S.); t-shiba@ihwg.jp (T.S.); yinlu.hope1020@gmail.com (L.Y.); 2 Department of Physical Therapy, School of Health Sciences, International University of Health and Welfare, Otawara 324-8501, Japan; sawaya@ihwg.jp (Y.S.); n-tamaki@ihwg.jp (T.H.); ishizaka@ihwg.jp (M.I.); 3 Nishinasuno General Home Care Center, Department of Day Rehabilitation, Care Facility for the Elderly “Maronie-en”, Nasushiobara 329-2763, Japan; shuntaro.tsuji.0614@gmail.com; 4 Department of Geriatric Medicine, School of Medicine, International University of Health and Welfare, Chiba, Narita 286-8686, Japan

**Keywords:** gait speed, activities of daily living, sarcopenia, older adults, long-term care

## Abstract

*Background and Objectives*: Early assessment interventions are recommended for older adults with sarcopenia. Gait speed in older adults considerably decreases activities of daily living (ADL). However, the association between ADL and gait speed in older adults with sarcopenia has not yet been fully elucidated. This study aimed to clarify the association between walking speed and ADL in older adults with sarcopenia. *Materials and Methods*: A total of 72 older adults with sarcopenia who required support or care under Japan’s long-term care insurance system were included. Correlation and multivariate analyses were performed to examine the association between walking speed and ADL performance. A receiver operating characteristic analysis was used to evaluate the discrimination power of gait speed for ADL independence. *Results*: Gait speed was significantly and positively correlated with the Barthel Index scores for the men and women. ADL were independently and significantly associated with walking speed in the multivariate analysis. The threshold for gait speed that distinguished ADL independence in older adults with sarcopenia was 0.76 m/s (area under the curve = 0.75, sensitivity 72.7%, specificity 74.0%). *Conclusions*: Decreased gait speed in older adults with sarcopenia was associated with decreased ADL. Gait speed had high discriminatory power in identifying ADL independence. This indicates that an assessment intervention for gait speed in older adults with sarcopenia may have high clinical utility.

## 1. Introduction

Sarcopenia is defined as an age-related decline in skeletal muscle mass and muscle strength [1]. Sarcopenia has been reported to be associated with decreased activities of daily living (ADL) [2] and increased risks of rehospitalization [3] and mortality [4], indicating the importance of nutritional and exercise interventions for its management [5,6]. In Japan, the overall incidence of sarcopenia among community-dwelling older adults is approximately 14.1%. However, the decline in skeletal muscle mass progresses with age, with prevalence rates of approximately 5.2% among individuals aged 65–69 years, and 40.8% among those aged ≥80 years, demonstrating a clear increasing trend [7]. Furthermore, sarcopenia is highly prevalent among older adults requiring long-term care, with a prevalence of approximately 56.3% [8] and a new-onset rate of 13.0% after 6 months, underscoring the importance of early assessment and intervention [9]. Therefore, facilities that provide medical and nursing care and assessment interventions to prevent decline in ADL are essential for the continuation of independent living for older adults with sarcopenia. Furthermore, recent studies have reported that depression is associated with care dependency among older adults with sarcopenia [10], and that obesity is associated with increased mortality risk [11]. An aging society is one of the most important public health challenges in developed Asian countries because the number of people requiring long-term care is rising, and the need to promote the extension of healthy life expectancy is growing [12,13,14].

In previous studies, factors associated with decreased ADL in older adults included low nutritional status [15], grip strength [16], skeletal muscle mass index (SMI) [17], and decreased walking speed [18]. Improvement in walking speed through rehabilitation is associated with enhanced ADL [19,20]. However, previous studies have focused on community-dwelling older adults, leaving the factors related to ADL and the characteristics of older adults with established sarcopenia unclear. Identifying the features of sarcopenia that are closely associated with ADL decline could aid in rehabilitation management and enable earlier intervention for older adults requiring long-term care.

Therefore, we hypothesized that ADL and gait speed would be reduced in older adults with sarcopenia. This study aimed to clarify whether gait speed can be used to identify impaired ADL in older adults with sarcopenia.

## 2. Materials and Methods

### 2.1. Ethical Considerations

This study is a single-center cross-sectional study involving older adults receiving day care services. This cross-sectional study was conducted between March 2023 and September 2024. The study protocol was approved by the Ethics Review Board of our facility (approval numbers: 21-Io-22-3 and 17-Io-189-7). All the participants (or their family members) provided written informed consent. This study was conducted in accordance with the principles of the Declaration of Helsinki.

### 2.2. Study Participants

This cross-sectional study enrolled 208 individuals assessed as requiring support or care under the Japanese long-term care insurance system and were utilizing day rehabilitation facilities for older adults [21]. Their body composition was measured.

We included 72 older adults who met the inclusion and exclusion criteria in the study analysis. The inclusion and exclusion criteria for this study were established by referencing the subject criteria used in previous studies [22], with the additional criterion of excluding non-sarcopenia cases. The exclusion criteria were individuals (1) younger than 65 years, (2) older than 100 years, (3) requiring walking assistance, (4) who did not present with sarcopenia, and (5) with missing data.

### 2.3. Measurements

#### 2.3.1. Sarcopenia Diagnosis

Gait speed was measured twice at a normal walking pace between 3 and 8 m (5 m) on an 11 m walking path; the average value was used as the representative value.

Grip strength was assessed twice in the right and left hands in the sitting position using a grip strength meter (TKK5401 Grip-D; Takei Kiki, Niigata, Japan). The maximum value was considered the representative value.

Skeletal muscle mass was measured in the sitting position using a body composition analyzer (InBodyS10; InBody, Seoul, Republic of Korea). Sarcopenia diagnostic criteria were defined according to Asian Working Group for Sarcopenia (AWGS) 2019 as appropriate for Asians [23]. For men, low muscle strength was defined as grip strength < 28 kg, low physical function as walking speed < 1.0 m/s, and low skeletal muscle mass as SMI < 7.0 kg/m^2^. For women, low muscle strength was defined as grip strength < 18 kg, low physical function as walking speed < 1.0 m/s, and low skeletal muscle mass as SMI < 5.7 kg/m^2^. We classified the participants into the sarcopenia (severe sarcopenia and sarcopenia) and non-sarcopenia groups.

#### 2.3.2. ADL Assessment

The Barthel Index (BI) was used for ADL assessment; the score ranges from 0–100 and a higher BI score indicates less dependence. The BI has been reported as a reliable ADL assessment tool [24]. The inter-rater reliability was ICC = 0.98 (95% confidence interval: 0.97–0.98), indicating that this is a highly reliable ADL assessment tool [25].

#### 2.3.3. Other Covariates

Age, sex, and height data were obtained from the facility’s medical records. Nutritional status was determined using the Mini Nutritional Assessment Scale-Short Form (MNA-SF), which is recommended for use with older adults [26]. The MNA-SF scores were classified as 0–7 (malnutrition), 8–11 (at risk for malnutrition), and 12–14 (no malnutrition).

### 2.4. Statistical Analyses

Pearson’s and Spearman’s rank correlation coefficients were used to analyze the factors related to ADL. A multiple regression analysis was performed using gait speed as the independent variable to examine its relationship with the BI score, the dependent variable. This study investigated which indicators—grip strength, walking speed, and SMI—are associated with ADL. The MNA-SF was included as a control variable, along with gender, age, and BMI, as it may act as a confounding factor for both ADL and sarcopenia [9].

A receiver operating characteristic curve was created to determine the threshold for gait speed to identify ADL independence (BI = 100) and the area under the curve (AUC). Sensitivity and specificity were calculated using the Youden Index to determine the optimal threshold for AUC values: >0.9 (high precision); 0.7–0.9 (moderate precision); and <0.7 (low precision) [27].

Statistical analyses were performed using the Statistical Package for the Social Sciences, Version 25 (IBM Corp., Armonk, NY, USA). Significance was set at *p* < 0.05. A power analysis was performed using G*Power version 3.1.2.1.

### 2.5. Sample Size

The power analysis utilized a bivariate normal model with a *t*-test, yielding a power of 0.99 using an effect size of 0.47 for walking speed and BI, an alpha error probability of 0.05, and a sample size of 72.

## 3. Results

**Figure 1:**
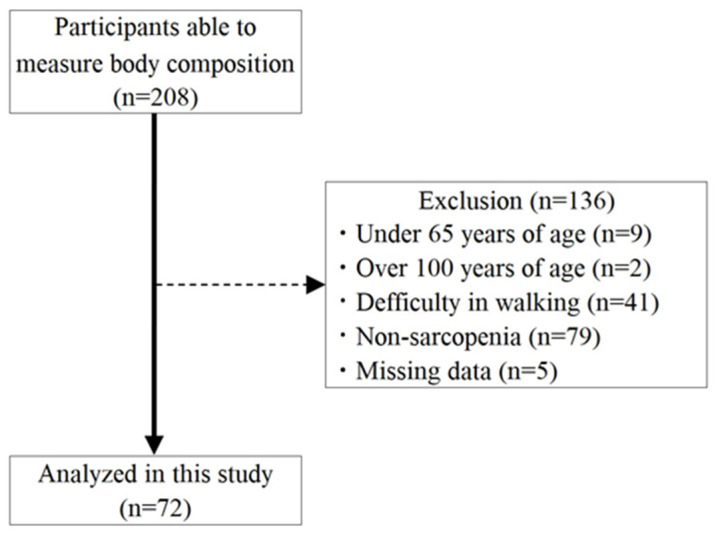
Selection process flowchart for the study participants.

Figure 1 presents the flowchart of those enrolled and the 72 older adult study participants. Table 1 is a summary of the basic attributes, ADL, grip strength, walking speed, SMI, and nutritional status of the study participants. Since the proportion of missing values in this study is less than 10%, the listwise deletion method was adopted.

Table 2 lists the factors related to ADL. The correlation analysis revealed significant positive correlations between BI, grip strength (r = 0.26), and walking speed (r = 0.47). A significant positive correlation was observed between BI, grip strength (r = 0.31), and walking speed (r = 0.50) for both men and women. The BI score and gait speed for women were significantly positively correlated (r = 0.45).

Table 3 presents the results for the relationship between the walking speed and ADL performance. The multiple regression analysis revealed a significant association between walking speed (β = 0.45, *p* < 0.001, R^2^ = 0.29) and ADL independence.

**Figure 2:**
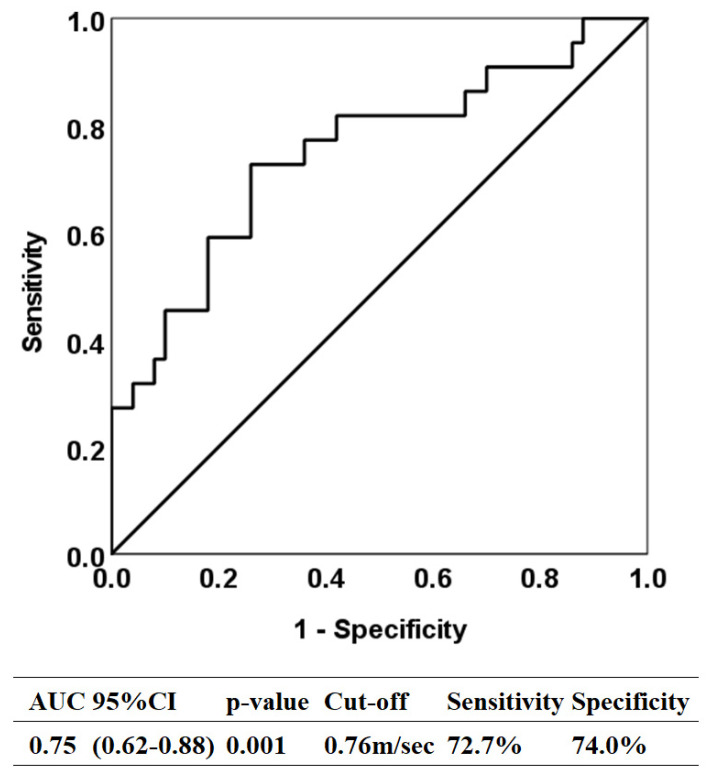
Receiver operating characteristic curve analysis to identify independence for activities of daily living owing to gait speed. AUC, area under the curve; CI, confidence interval.

Furthermore, 22 participants (30.6%) had a BI score of 100. The optimal threshold for walking speed to identify ADL independence (BI = 100) was 0.76 m/s (AUC = 0.75, 95% CI: 0.62–0.88, sensitivity 72.7%, specificity 74.0%) (Figure 2). Only one point at which the Youden Index reached its maximum value was identified.

## 4. Discussion

In this study, we identified factors associated with ADL of older adults with sarcopenia. In addition, we determined the discriminatory power of normal gait speed for identifying ADL independence. To the best of our knowledge, this was the first study to evaluate the association between gait speed and ADL in older adults with sarcopenia.

We found a significant positive correlation between gait speed and ADL for both men and women. Gait speed SMI, and grip strength are key components of sarcopenia assessment and are expected to be lower compared to other groups. This study revealed that, among sarcopenic older adults with reduced gait speed, SMI, and grip strength, gait speed in particular is closely associated with ADL. Gait speed in older adults is associated with balance function [28] and muscle mass asymmetry [29]. The group with slower gait speed had a higher fall rate than the group with higher gait speed [30]. A previous study reported a correlation between gait speed and ADL, low walking speed, and increased risk of death [31]. Furthermore, because walking speed is associated with age-related frailty [32], an indirect association may be possible. In a population of older adults with care needs, a decline in normal gait speed can indicate worsening of independent care capacity, suggesting the need for early assessment interventions for gait speed [33]. The results of this study support previous findings showing that decreased normal gait speed is associated with decreased ADL. Interestingly, this study revealed that gait speed, and not skeletal muscle mass, influenced ADL status in older adults with sarcopenia. Previous reports show an association between skeletal muscle mass and ADL [34]. This may be owing to differing ages of the participants in this study compared to those in previous studies; in addition, the participants in this study sarcopenic population with low skeletal muscle mass, indicating that gait speed, a measure of physical function, may have a profound effect on ADL. This novel study clarified the relationship between ADL and normal gait speed in older adults with sarcopenia. Nutrition and exercise have been reported to improve walking speed and SMI in older individuals with sarcopenia [35]; however, no studies have examined their effects on ADL. Therefore, future rehabilitation protocols should incorporate ADL assessment and targeted intervention.

Recent studies indicate that low physical performance, defined as a walking speed ≤ 0.8 m/s, is a strong predictor of functional impairment in individuals aged 60 years and older [36]. In populations with a high proportion of individuals with disabilities, a cutoff of 0.6 m/s may serve as a useful threshold for identifying the risk of further functional decline in older adults who already have functional impairments [37]. In this study, we postulated a new reference value for walking speed to identify ADL in older adults with sarcopenia. The results indicated that the optimal gait speed threshold to identify ADL independence was 0.76 m/s. For individuals aged 75 years and older, reduced walking speed (<0.7 m/s) is a predictor for hospitalization, need for a caregiver, and new falls [38]. Previous studies indicate an average walking speed of 0.70 ± 0.18 m/s among sarcopenic older requiring care [39]. Given that this study population consists of older sarcopenic adults, a walking speed cutoff of 0.76 m/s is considered appropriate. The results of this study showed sufficient discrimination power for identifying ADL independence, which suggest that the thresholds obtained are reasonable indicators; indicating older adults with sarcopenia-reduced skeletal muscle mass can be independent in ADL if their walking speed is maintained at 0.76 m/s.

This study has a few limitations. First, it was cross-sectional; therefore, the causal relationship between ADL and decreased gait speed remains unknown. To clarify causality, longitudinal follow-up studies of participants are desirable, along with longitudinal validation of the gait speed cutoff value. Second, regarding the reliability of the gait speed cutoff, single-center bias and preexisting conditions may affect its robustness. Frail older adults often have multiple comorbidities, making it difficult to adjust for the effects of these conditions [40]. Considering the skewed distribution of participants’ ADL independence levels, future research would benefit from strategies for gait classification under conditions of class imbalance and limited sample size [41]. Additionally, future research should utilize 3D motion analysis systems for gait speed assessment to employ more reliable measurement tools [42]. Third, this study did not include frailty indices or the Charlson Comorbidity Index [43,44]. Including these indices may improve the robustness of findings. Moreover, this approach does not adequately capture the contribution of cognitive-behavioral factors to instrumental activities of daily living and independence; using a broader battery of tests would improve functional characterization [45]. Furthermore, since DEXA/CT/MRI [46], creatinine excretion [47], and isokinetic dynamometry are also used to assess or evaluate its associations [48], it would be valuable for future research to investigate correlations with these indicators as well. Finally, walking speed is recognized as a key functional indicator in the AWGS 2025 Consensus and an outcome of muscle function decline [1]. Future research should examine this according to various sarcopenia criteria [49].

## 5. Conclusions

In conclusion, we found that a decreased normal gait speed in patients with sarcopenia was associated with decreased ADL. Furthermore, normal gait speed was highly discriminative of ADL independence. This indicates that a clinical assessment intervention for gait speed can be highly useful for maintaining independent ADL in older adults with sarcopenia.

## Figures and Tables

**Table 1:** Overall participant characteristics.

	Total (n = 72)
Sex (Women: %)	31.0%
Age (years)	82.1 ± 6.7
Height (cm)	157.2 ± 8.7
Weight (kg)	53.0 ± 8.2
BMI (kg/m^2^)	21.4 ± 2.9
Barthel Index (score)	90 (83.8–100)
Grip strength (kg)	18.8 ± 6.0
Gait speed (m/s)	0.7 ± 0.2
SMI (kg/m^2^)	5.9 ± 0.7
Malnutrition (%)	26.4%
At risk of malnutrition (%)	47.2%
Non-malnutrition (%)	26.4%

The data are denoted as mean ± standard deviation or median (1st–3rd quartiles). BMI, body mass index, SMI, skeletal muscle mass index. SMI = skeletal muscle mass (kg)/height^2^ (m^2^); BMI = weight (kg)/height^2^ (m^2^).

**Table 2:** Correlations between variables.

	Barthel Index (Score)	Age (Years)	BMI (kg/m^2^)	Grip Strength (kg)	Gait Speed (m/s)	SMI (kg/m^2^)	MNA-SF (Score)
(Total)							
Barthel Index (score)	−	−0.06 (0.633)	0.01 (0.923)	0.26 (0.028)	0.47 (<0.001)	0.13 (0.284)	0.08 (0.526)
Age (years)	−	−	−0.07 (0.575)	−0.15 (0.210)	0.00 (0.993)	0.00 (0.972)	0.13 (0.281)
BMI (kg/m^2^)	−	−	−	0.02 (0.857)	−0.11 (0.360)	0.16 (0.189)	0.40 (0.001)
Grip strength (kg)	−	−	−	−	0.36 (0.002)	0.60 (<0.001)	0.21 (0.074)
Gait speed (m/s)	−	−	−	−	−	0.21 (0.076)	0.12 (0.314)
SMI (kg/m^2^)	−	−	−	−	−	−	0.29 (0.014)
MNA-SF (score)	−	−	−	−	−	−	−
(Men)							
Barthel Index (score)	−	0.10 (0.549)	0.01 (0.946)	0.26 (0.097)	0.49 (0.001)	−0.01 (0.948)	0.15 (0.354)
Age (years)	−	−	−0.16 (0.311)	−0.20 (0.208)	0.13 (0.0.433)	0.17 (0.301)	0.15 (0.348)
BMI (kg/m^2^)	−	−	−	−0.01 (0.926)	−0.11 (0.489)	0.28 (0.072)	0.29 (0.063)
Grip strength (kg)	−	−	−	−	0.30 (0.058)	0.05 (0.744)	0.19 (0.244)
Gait speed (m/s)	−	−	−	−	−	0.03 (0.832)	0.13 (0.429)
SMI (kg/m^2^)	−	−	−	−	−	−	0.21 (0.211)
MNA-SF (score)	−	−	−	−	−	−	−
(Women)							
Barthel Index (score)	−	−0.20 (0.282)	0.02 (0.934)	0.33 (0.074)	0.45 (0.011)	0.17 (0.348)	−0.09 (0.630)
Age (years)	−	−	0.03 (0.858)	−0.08 (0.662)	−0.15 (0.411)	−0.06 (0.752)	0.12 (0.526)
BMI (kg/m^2^)	−	−	−	0.17 (0.354)	−0.10 (0.580)	0.32 (0.081)	0.47 (0.008)
Grip strength (kg)	−	−	−	−	0.24 (0.201)	0.28 (0.132)	0.18 (0.336)
Gait speed (m/s)	−	−	−	−	−	0.02 (0.914)	−0.05 (0.783)
SMI (kg/m^2^)	−	−	−	−	−	−	0.32 (0.0076)
MNA-SF (score)	−	−	−	−	−	−	−

BMI, body mass index; MNA-SF, Mini Nutritional Assessment Scale-Short Form; SMI, skeletal muscle mass index. SMI = skeletal muscle mass (kg)/height^2^ (m^2^); BMI = body weight (kg)/height^2^ (m^2^); values between parentheses indicate *p*-values.

**Table 3:** Factors related to activities of daily living according to the multiple regression analysis.

	β	B	95% CI	*p*-Value	VIF
Gait speed (m/s)	0.45	19.53	(9.54–29.52)	<0.001	1.18
Sex (Men: 0, Women: 1)	0.21	4.49	(−4.09–13.06)	0.300	3.68
Age (years)	−0.02	−0.04	(−0.38–0.31)	0.838	1.06
BMI (kg/m^2^)	0.11	0.42	(−0.44–1.27)	0.3372	1.25
Grip strength (kg)	0.27	0.49	(−0.07–1.04)	0.0841	2.19
SMI (kg/m^2^)	0.04	0.65	(−4.85–6.14)	0.8148	3.22
MNA-SF (score)	−0.04	−0.16	(−1.14–0.81)	0.739	1.28

β, standardized regression coefficient; B, unstandardized regression coefficient; CI, confidence interval; BMI, body mass index; MNA-SF, Mini Nutritional Assessment-Short Form; SMI, skeletal muscle mass index; VIF, variance inflation factor. SMI = skeletal muscle mass (kg)/height^2^ (m^2^); BMI = weight (kg)/height^2^ (m^2^).

## Data Availability

The data presented in this study are available from the corresponding author only upon reasonable request, due to ethical restrictions.
